# Scientific and Regulatory Perspectives in Herbal and Dietary Supplement Associated Hepatotoxicity in the United States

**DOI:** 10.3390/ijms17030331

**Published:** 2016-03-03

**Authors:** Mark I. Avigan, Robert P. Mozersky, Leonard B. Seeff

**Affiliations:** 1 Office of Pharmacovigilance and Epidemiology, Center for Drug Evaluation and Research, Food and Drug Administration, 10903 New Hampshire Avenue, Silver Spring, MD 20993, USA; mark.avigan@fda.hhs.gov; 2 Office of Dietary Supplement Products, Center for Food Safety and Applied Nutrition, 5100 Paint Branch Parkway, College Park, MD 20740, USA; robert.mozersky@fda.hhs.gov; 3 6403 Hillmead Rd, Bethesda, MD 20817, USA

**Keywords:** US Food and Drug Administration, regulation, Dietary Supplement Health and Education Act (DSHEA), herbal supplement epidemiology, drug induced liver injury, herbal supplement contamination, herbal supplement adulteration, challenges in assessing herbal hepatotoxicity

## Abstract

In the United States (US), the risk of hepatotoxicity linked to the widespread use of certain herbal products has gained increased attention among regulatory scientists. Based on current US law, all dietary supplements sold domestically, including botanical supplements, are regulated by the Food and Drug Administration (FDA) as a special category of foods. Under this designation, regulatory scientists do not routinely evaluate the efficacy of these products prior to their marketing, despite the content variability and phytochemical complexity that often characterizes them. Nonetheless, there has been notable progress in the development of advanced scientific methods to qualitatively and quantitatively measure ingredients and screen for contaminants and adulterants in botanical products when hepatotoxicity is recognized.

## 1. Introduction

Regulatory scientists in many countries across the world have become increasingly aware of cases of clinically serious hepatotoxicity that are causally linked to a number of herbal and botanical products marketed as dietary supplements [1,2,3]. In the United States (US), concern surrounding this adverse effect has gained prominence, as there has been a steady rise of usage of dietary supplements over the last few decades by demographically diverse groups of consumers [4,5,6]. Highlighting this concern, an ongoing prospective multi-center study by the National Institutes of Health (NIH)-sponsored Drug-Induced Liver Injury Network (DILIN) has determined that 15.5% of the domestic hepatotoxic events leading to enrollment were causally associated with dietary supplements and herbal products [7]. Over the course of the study period (2004–2013), herbal-related drug-induced liver injury (DILI) increased from 7% to 20% [8]. The perspectives that are developed in this article are primarily connected to current laws and regulation governing the marketing of all dietary supplements in the US. It is important to recognize that dietary supplements comprise a broad category of products that are not prescription or over-the-counter drugs. These include not only herbal and botanical products but also vitamins, minerals, amino acids, and certain other non-prescription products that supplement the diet.

The Food and Drug Administration (FDA) does not routinely perform pre-marketing safety evaluation of dietary supplements and does not register all marketed supplements. Its regulatory scientists often do not have available for review product ingredient content or exposure outcome measurements at the onset of an investigation that is tasked to assess a post-marketing adverse event, such as supplement-associated liver injury. Compounding this challenge in the evaluation of herbal-related safety signals, FDA scientists together with non-agency regulatory analysts must take into account the content variability and phytochemical complexity that typically characterizes different preparations and batches of many botanical products [9,10]. Despite these hurdles, there has been notable progress in the development of advanced methods to qualitatively and quantitatively measure ingredients and screen for contaminants and adulterants in botanical products [9,11]. In the future, with a more routine application of such methods in conjunction with the emergence of accessible product-specific fingerprint databases, the scientific authentication of botanical products and identification of candidate compounds, adulterants or contaminants that may cause hepatotoxicity will enhance the reliability, quality and safety of these commercial supplements.

### Global Challenges in Evaluating Herbal Product Risk for Liver Toxicity

Herbals/botanicals utilized for medicinal purposes consist of two broad categories. The first are natural products derived from plants (flowers, stems, roots, leaves, berries, seeds) and barks of trees; their use dates back centuries [3]. Herbs were used in ancient times to “promote health” by strengthening the body’s ability to fend off or deal with illnesses, as well as to treat pain and injuries. Although generally used as a single product, the actual herbs consist of a variable number of chemical constituents, a fact obviously not appreciated until the advent of modern chemistry. However, the chemical constituents of many remain unknown or vary in the same product depending on the time of the year of growth, geographic elevation, or geographic location where grown. Of concern is that occasional products, both those especially cultivated and those growing in the wild, have been found to be contaminated with pesticides, heavy metals such as lead, mercury and arsenic, microbial agents and mycotoxins [12,13,14,15,16,17,18,19,20,21].

As many as 90% of the African population, 70% of the Indian population and 40% of the Chinese population continue to depend on and utilize herbals/botanicals for general healthcare [22]. Moreover, they have been and continue to be a rich source for creating important new pharmaceutical drugs; indeed, well over 100 key drugs have been derived from herbals/botanicals in the past. Among these are acetylsalicylic acid (aspirin), quinine, morphine and other opioids, digitalis, atropine, vinblastine, vincristine, colchicine, ephedrine, papaverine, reserpine, taxol, and many more. Currently, there is a vigorous and ongoing effort to identify herbals that might yield chemicals with medicinal properties, especially for the treatment of cancers [23].

In the mid-20th century, with the growing appeal of a holistic way of life, a second category of herbal products began to enter the field, namely those produced by commercial entities. Interest by the public now was to use herbals not only to “treat” actual disease or symptoms, either together with prescription drugs (‘complementary” medicine) or on their own (“alternative” medicine), but even more commonly to improve quality of life and wellbeing, support “natural” healing, boost the immune system, or in order to lose weight or enhance muscle growth during bodybuilding. These commercial products generally consist of multiple herbal constituents bundled together, sometimes tied to an assumption that if an individual product is thought useful, a combination will be even more effective. The number of such products can range from two to more than 10, but they do not necessarily complement one another. Herbals differ from pharmaceutical drugs in a vital way; whereas prescription drugs are manufactured in a consistent and chemically standardized fashion, the production of herbals, whether traditional crude or commercial, cannot duplicate the same manufacturing procedures to ensure content identity. In some instances the ingredients have been found to differ from that described on the product label [24]. Moreover, the concentrations of ingredients have been found to be inconsistent, with variations detected among lots of production [25]. In addition, surreptitious product adulteration with prescription drugs, a deceptive practice, has been documented on multiple occasions. As noted below, corticosteroids, sildefanil, benzodiazepines, and diclofenac are among the many agents that have been identified in adulterated supplements [26,27,28].

## 2. Trends of Dietary Supplement Use in the US

From an epidemiological perspective, an important factor that drives the total burden of domestic cases of idiosyncratic DILI are the overall levels of exposure in the nation’s population to agents causally associated with these adverse events. In tandem with the observation that dietary supplements are responsible for an increasing percentage of all domestic cases of drug or supplement-induced liver injury is the presence of rich national usage data derived from a number of governmental sources and trade-associations [29,30,31,32,33,34,35,36,37,38,39,40]. These indicate that there has been a steadily rising consumption of dietary supplements by US residents over the last few decades. The National Center for Health Statistics (NCHS) has tracked trends in usage of these products through a periodically administered, nationally representative cross-sectional survey entitled the National Health and Nutrition Examination Survey (NHANES) [29,30,36,37]. This survey of domestic residents consists of varying questionnaires on a variety of nutritional and health-related topics. Incorporating in-person household interviews, the survey utilizes a cluster sampling design with fractionations based on region, neighborhoods and other multi-staged criteria. Since the Dietary Supplement Survey questionnaire is administered every few years, NHANES is able to ascertain trends of usage of vitamins, minerals herbals and other supplements over time. Between the 1988–1994 (NHANES III) and the 2003–2006 survey cycles, the age-adjusted prevalence of dietary supplement usage in US adult residents in the past 30 days rose from 42% to 53% [30]. In the 2003–2006 survey, supplement use was higher in females than in the males [30]. Moreover, demographic stratification revealed that the highest concentration of overall supplement use was among older non-Hispanic whites, in particular those with more than a high school education. Twenty percent of surveyed individuals reported botanical ingestion, the majority using formulations of combination products with other botanical or non-botanical components. The highest use was in older adults, peaking in the 51–70 year age group.

When stratified by reasons for use, dietary supplements possessing claims of improving cognitive and mental functions or preserving health were heavily used by older age groups, whereas younger age groups were the most frequent users of agents meant to increase muscle strength (Male (M) > Female (F)) or reduce weight (F > M) [36]. Age-related reasons for the use of supplements have been further explored in a more recent 2007–2010 NHANES survey [36]. Older adults tended to use supplements chronically to maintain long-term organ specific functions (e.g., preservation of bone, heart, prostate), whereas younger adults were prone to use supplements for short-term gains, such as enhancing energy or boosting immune function. Both NHANES III and the National Health Interview Study (a nationally representative cross-sectional study developed by the NCHS and administered by the US Census Bureau; surveys performed in 2002 and 2007) [37,38] found that individuals who reported using botanicals were likely to also be users of prescription and over-the-counter drugs and often claimed to have pre-existing medical conditions. They tended to conceal their botanical use from their physicians and other health-care providers [37,38], a point of considerable concern since a number of herbal agents are associated with significant herbal-drug interactions [41,42].

Recent surveys of US military personnel reveal substantial levels of dietary supplement use to enhance body-building, boost energy or induce weight loss [39]. In a 2001–2008 survey of 115,382 active-duty Reserve and National Guard personnel (component of the 21-year longitudinal Millennium Cohort Study) [39], 46.7% of the participants reported use of at least one dietary supplement in the past 12 months. Among all the respondents, 17.3% reported use of bodybuilding supplements (M > F), 38.0% reported use of energy supplements (M > F) and 19.4% reported use of weight-loss supplements (F > M). Deployment experience, young age and “problem-drinking” of alcohol were more likely to characterize individuals who had used any of these three types of supplements compared with those individuals who had not used them. In a different survey of 576,284 active-duty military personnel performed by the Department of Defense in 2005 [40], 58.3% of male respondents and 71.4% of female respondents reported use of a dietary supplement at least once per week. Among the respondents, 20.5% reported using body building supplements (M > F), 18% reported using weight-loss supplements (F > M), 11.7% reported using herbal products, (M = F), and 8.4% reported using performance enhancing supplements (M > F). Remarkably, among the respondents who used dietary supplements, 30.7% reported daily use and 12.8% reported use two or more times/day. Moreover, only 37% of them informed their physicians of this use. Interestingly, females and older personnel were more likely than their counterparts to report use of these products to health-care professionals.

Based on these survey data, there is strong evidence for the widespread utilization of dietary supplements, including botanical agents in the US population. These products are taken for different purposes, including bodybuilding, energy boosting and weight loss. With this broad unsupervised usage, it is not surprising that varying types of DILI could result through a diverse set of hepatotoxicity mechanisms, including excessive dosing of some plant-derived chemicals, or ingestion of other hepatotoxic contaminants or adulterants. In some instances, they likely reflect idiosyncratic forms of liver injury.

## 3. Regulatory History and Framework for the Legal Marketing of Herbals and Dietary Supplements in the US

### 3.1. Laws, Guidance and Regulatory Framework

To ensure that the American public has access to safe foods and drugs, the US Congress enacted legislation to create the FDA for regulating the food and drug industries and, over the course of time, established a number of relevant laws [43]. In 1906, Congress introduced the “Pure Food and Drug Act”, a law that allowed government inspectors to prevent adulterated foods and drugs from entering interstate commerce. In 1938, responding to the tragic death of more than 100 individuals who had ingested an adulterated drug, Congress passed the Food, Drug and Cosmetic Act (FD&C Act). The Act defined a drug as a “substance that is intended for use in the diagnosis, cure, mitigation, treatment, or prevention of disease in man or other animals”. Additionally, the law required drugs to be proven safe before marketing, to have safe tolerance levels for unavoidable poisonous substances (*i.e.*, pesticides), authorized factory inspections, and allowed for court injunctions if imposed penalties were not properly applied. For example, if the FDA seized a drug and the company contested the seizure, a court could render a decision on the appropriateness of the seizure. The Kefauver-Harris Drug Amendments were enacted in 1962, and remains one of the most important set of laws governing drug marketing. The law required that drugs must be shown to be both effective and safe before being approved for use. When the law was passed, vitamins and minerals were considered over-the-counter drugs, and therefore were regulated as drugs.

In 1994, Congress passed the Dietary Supplement Health Education Act (DSHEA), defining “dietary supplement” and “new dietary ingredient (NDI)” [44]. The Act established specific labeling requirements and reclassified vitamins and minerals as dietary supplements. The DSHEA defines a dietary supplement as “a product other than tobacco intended to supplement the diet: a vitamin, a mineral, herbs or other botanical, an amino acid, a dietary substance for use by man to supplement the diet by increasing the total dietary intake, or a concentrate, metabolite, constituent, extract, or a combination of any of the aforementioned ingredients”. It also stipulates that a dietary supplement is not meant to replace a meal. Lastly, dietary supplements have to be administered in one of the following forms: a tablet, gel cap, capsule, softgel, powder, or liquid. The DSHEA allows manufacturers to market their dietary supplement products without having to receive approval from the FDA. This means that the manufacturing company need not prove the efficacy of their product and also that it is itself responsible for the product safety. This is in marked contrast with pharmaceutical drugs, which cannot be marketed until sponsors demonstrate that their product is both effective and safe.

Regarding the NDI, Congress stipulated that all dietary supplements sold before 1994 were to be considered safe and could therefore remain on the market without the manufacturer having to file an NDI notification [44]. The FDA must receive notification of any supplement with a new ingredient(s) marketed after 1994, showing information regarding the manufacturer, the manufacturing process, and the product’s safety. The NDI notification must be received 75 days before marketing of the product. On or before day 75, the FDA must then notify the company regarding its assessment of the NDI application. Later, on day 90, the FDA can publish most of the information conveyed in the notification, while excluding trade secrets and other proprietary information. The manufacturer may choose to market its dietary supplement, even if it had received a letter from the FDA indicating that the NDI notification was inadequate. However, the FDA then has the prerogative, based on its evaluation, to take a regulatory action against the manufacturer.

In 2006, Congress passed the Dietary Supplement and Non-Prescription Drug Protection Act [45]. Prior to its passage, manufacturers of dietary supplements and over-the-counter drugs were not required to notify FDA of adverse events regarding their products. After passage of this law, the requirement to report serious adverse events came into being.

In 2007, the FDA published the final rule regarding current Good Manufacturing Practices (cGMP) for firms manufacturing dietary supplements [46]. This regulation informed companies that they were required to maintain quality standards to ensure that the dietary supplement(s) they marketed were safe.

### 3.2. Path for Approval of Herbal Products by the FDA

Prior to the emergence of pharmacologic agents in the 19th century, all medical ailments and diseases were treated with traditional botanical products [47,48]. Subsequently and until the mid-1980’s, botanicals continued to be particularly important since they were, in fact, the source for production of most of the new pharmacologic drugs [49,50]. As noted above, key pharmaceuticals were developed from herbals and botanicals, many predating the institution of regulatory laws. Thereafter, while developers of pharmacologic drugs were required to subscribe to FDA regulations and perform clinical trials to determine drug efficacy, herbal dietary and botanical supplements were excluded from this requirement [44]. Because, however, botanicals continue to be a potentially rich source for the production of new drugs, and especially drugs to treat cancers, the FDA has established a review team to develop guidelines for the marketing and regulating of botanical products as over-the-counter drugs, to subscribe to the same level of stringency as is expected of pharmaceutical drugs [51]. These guidelines were published in 2004 as “Guidance for Industry: Botanical Drug Products” [52]. The FDA received over 400 NDI applications for new botanicals between 2004 and 2013 [52]. While most were permitted to enter phase 2 clinical trials, only two have thus far received FDA approval, Veregen (sinecatechins) in 2006 and Fulzaq (crofelemar) in 2012 [51]. A number of the remaining products are currently undergoing phase 3 trials.

## 4. Manufacturing Dietary Supplements

Dietary supplements contain many different types of ingredients. Botanicals/herbs have unique dietary ingredients because they are grown in soil. Different parts of the plant, stem, leaf, or root provide different quantities of an active ingredient. Active ingredients in plants can be affected by soil type, climate and time of harvest. Depending on the product, the company will determine which steps will be included in the manufacturing process. These steps include adhering to cGMP, authenticating the plant, ensuring the presence of the active ingredient, and overseeing quality control methods to guarantee safety.

The first step involves following cGMP for ingredients harvested from the ground. This requirement increases the likelihood that a contaminant, such as a pesticide or heavy metal, is at its lowest level at the time of harvest. The United States Department of Environmental Protection Agency (EPA) [53] and the FDA have written guidance detailing the amount of these adulterants that the plant may contain [54].

Step two involves accurately identifying the plant so that the correct plant is incorporated into the product. A company can use any one of the following techniques: macroscopic, organoleptic, microscopic, and/or chromatographic evaluation. One technique under development—DNA fingerprinting—involves comparison of the DNA of the plant inserted into the product with reference genes from an authenticated plant. This step also involves proper identification of any synthetic compounds in the product. A company may use any one of the following techniques for this purpose: gas chromatography, liquid chromatography, and absorption spectrophotometry.

Step three involves quality assurances. Quality control measures depend on the dietary supplement and manufacturing process. Atomic absorption, spectrophotometry, inductive coupled plasma and neutron activation analysis can be used to detect heavy metals, pesticides, and other toxins. To determine if a product contains a toxic microorganism, samples of the product are taken and placed in a medium that supports their growth. Controlling temperatures prevents ingredient(s) from decomposing during manufacture. Other quality control measures include ensuring the absence of contaminants such as drugs, allergens, and/or foreign objects, and accurately recording the presence, amount, and types of ingredients on the label.

## 5. Methods Supporting Dietary Supplement Safety

Dietary supplement safety can be affected by one or more factors. The presence of multiple ingredients in a supplement increases the possibility that one ingredient may inhibit or promote the absorption of another. A particular ingredient might mask symptoms of a disease, as for example; consuming folate might mask a vitamin B_12_ deficiency that could lead to macrocytic anemia [55]. Manufacturing and standardizing procedures can vary among supplements and thus affect their safety; for example, kavalactones can be extracted from kava using a water or alcohol extract [56]. Water extracts of kava contain different concentrations of kavalactones than alcohol extracts. This, most likely, accounts for the fact that water extracts of kava have long been used in ceremonies without causing serious adverse events whereas alcohol extracts have led to serious hepatic injury [57,58]. Lastly, dietary supplements may contain one to several ingredient(s); pharmaceutical drugs, on the other hand, usually contain only one or two active ingredients. Should an adverse event occur from dietary supplements, the large numbers of ingredients in many of the products could hamper the ability to identify the specific harmful constituent(s).

## 6. Organizations Inside and Outside of the US Government Who Regulate, Track and/or Scientifically Analyze the Influence of Dietary Supplements in the United States

The *Center for Food Safety and Applied Nutrition (CFSAN)* in the FDA has primary regulatory responsibility over the legal domestic marketing of dietary and herbal supplements [59]. Relevant to the study of suspected hepatotoxicity linked to these agents are other expert US governmental and non-governmental organizations or groups who sustain dedicated efforts to analyze or document the contents, quality, or safety of dietary or botanical supplements. Some of them may collaborate with or provide scientific input to CFSAN. In a few instances, other governmental groups have regulatory or research functions that can directly or indirectly touch on the safety of dietary supplements marketed in the US. A brief description of some of these important stakeholders and resource groups is provided below.

### 6.1. Other US Government Organizations

#### 6.1.1. FDA Center for Drug Evaluation and Research (CDER)

Using a variety of chromatographic and spectroscopic tools, the Division of Pharmaceutical Analysis (DPA) within the Office of Testing and Research (OTR) in the Center for Drug Evaluation and Research (CDER) is equipped to comprehensively screen marketed products, including dietary supplements, for the presence of drug or controlled substance adulterants. Dietary supplements spiked with these agents cannot be legally marketed since all drug-containing products must be approved by the FDA before marketing and are subject to appropriate controls regarding consumer access. Since CDER has regulatory oversight over prescription drugs, it works closely with CFSAN in taking necessary regulatory measures when a safety issue emerges as a consequence of supplement adulteration with a drug(s) or an observed drug-supplement interaction. Cases of hepatotoxicity in which adulteration of dietary supplements was identified are described below.

#### 6.1.2. US Department of Agriculture’s Agricultural Research Service (USDA/ARS)

The Nutrient Data Laboratory in USDA’s Beltsville Human Nutrition Center has been developing a web-based Dietary Supplement Ingredient Database (DSID) in collaboration with the Office of Dietary Supplements (ODS) at the NIH. Thus far, this collaborative effort involving the National Health Service/Centers for Disease Control (NHS/CDC), the FDA, the National Cancer Institute (NCI) and the National Institute of Standards and Technology (NIST) has separately released ingredient analyses of formulations for adult multivitamins and minerals (DSID-1; April 2009), children multivitamins (DSID-2; March 2012) and ω-3 fatty acid supplements and pre-natal care multivitamins (DSID-3; March 2015). Currently, an initiative to reliably evaluate ingredients in commercial botanical products is underway as a pilot study that is evaluating contents in green tea supplements. It aims to evaluate the precision and accuracy of methods of analysis for ingredients of interest by testing representative and top-selling products; obtain estimates of content and variability for each of the separately measured catechin isomers and epimers, caffeine and other ingredients; identify options of how to translate analytic results into product label information; and plan the next botanical study.

#### 6.1.3. National Toxicology Program (NTP)/National Institute of Environmental Health Sciences (NIEHS)

This program provides toxicological analyses relevant to environmental or dietary exposures that may be toxic. It works closely with other agencies, including the National Center for Toxicological Research (NCTR) and the National Institute of Occupational Safety and Health (NIOSH) at the Centers for Disease Control (CDC) when there are exposures of common concern that prompt a toxicological investigation. The NTP Botanical Supplements Program has in its armamentarium advanced chromatographic, spectroscopic and other fingerprinting tools to qualitatively and quantitatively characterize the chemical and physical composition of dietary supplements that are referred for evaluation. In addition, it screens for the presence of metals, molds, and pesticides that may be toxic and can undertake rodent studies to identify toxic biological and pathological response effects using both short-term and long-term exposure protocols.

#### 6.1.4. US Federal Trade Commission (FTC)

This governmental body regulates the advertising of foods and dietary supplements. US law prohibits false advertising as well as deceptive practices. Because the FDA regulates product labeling, the agency must work with the Federal Trade Commission (FTC) to identify violations of product claims, as well as to ensure sponsor adherence to legal and regulatory standards in all forms of advertising.

#### 6.1.5. NIH Office of Dietary Supplements (ODS)

The Office of Dietary Supplements (ODS) was established by the NIH to create a knowledge base and develop scientific approaches to reach a full understanding of dietary supplements. This office has resources to conduct or support research on specific supplements, disseminate research results to the public, and promote education for their safe and effective use. With such a broad mandate, ODS collaborates with many other health agencies to promote these goals and develop database tools that are widely accessible.

#### 6.1.6. NIH Center for Complementary and Integrative Health (NCCIH)

Formerly referred to as the National Center for Complementary and Alternative Medicine (NCCAM), NCCIH is the US government’s lead agency for scientific research on health systems, practices and products that are not considered modalities in the mainstream of conventional medicine. The center has established a broad set of rigorous requirements to ensure the integrity of dietary supplement clinical research projects. These requirements include strict standards for ingredient evaluation and batch consistency in government-funded clinical trials of supplements to ensure the interpretability and generalizability of the study results.

#### 6.1.7. Environmental Protection Agency (EPA)

This agency regulates human pesticide tolerance levels for foods (including dietary supplements) and establishes standards for drinking water.

#### 6.1.8. US Customs and Border Protection

The border security agency in the Department of Homeland Security works together with the FDA to prevent entry into the US of imported products (including foods and dietary supplements) that do not meet domestic standards for marketability.

#### 6.1.9. US Government Accountability Office (GAO)

An independent nonpartisan agency that works for Congress, the Government Accountability Office (GAO) supports congressional oversight to improve Government operations through reviewing the effectiveness of Government programs, analyzing such programs on request, and offering options to Congress that may lead to the establishment of laws or acts. With respect to dietary supplements, the GAO has worked on FDA issues regarding safety of the products and on efforts to improve consumer understanding, having identified a lack of knowledge by consumers of their efficacy and safety, and of the interpretation of labels. GAO also works to protect consumers, an example being a report of deceptive marketing to the elderly of some herbal products [60].

### 6.2. Non-Government Organizations

#### 6.2.1. Dietary Supplement Manufacturers and the Food Industry: 

Under current US law, manufacturers and other sponsors of dietary supplements have a legal and regulatory responsibility to maintain product quality and safety, identify and characterize NDIs (described above) with a notification to FDA, label their products in conformity with legal requirements, adhere to cGMPs, and report serious adverse events to FDA’s MedWatch program. In this regard, the private sector is comprised of key stakeholders including product sponsors who are obligated to ensure the reliability and safety of their products.

#### 6.2.2. US Pharmacopoeia (USP)

USP is a non-profit organization that sets standards for the identity, strength, quality and purity of medicines, food ingredients and dietary supplements. The USP has developed a Dietary Supplements Compendium (DSC) that it revises with updates every three years. The DSC catalogues monographs with specifications of identity, strength, quality and purity for a number of dietary supplement and herbal products. These are not independently reviewed or authenticated by US regulatory authorities.

#### 6.2.3. Academic Centers and Projects that Study Pharmacognosy and Drug Safety [61]

There are a number of academic government and university-based programs in the US with faculty dedicated to the study of effects or outcomes related to dietary supplements and herbal products. In peer-reviewed studies, they are investigating ingredients as well as therapeutic outcomes in study subjects treated with these agents. As can be gathered from the partial description of organizations that contribute resources and expertise, this network of public and private entities plays a crucial role in risk assessment and management of dietary supplement exposure in the US. Because of the inherent complexities and variability of supplement and herbal product formulations as well as limitations in the FDA’s pre-marketing regulatory authority over these products, the importance of this network to provide resources in elucidating safety issues (e.g., hepatotoxicity) when they occur, and to appropriately respond in order to protect the public health, cannot be overstated.

## 7. Pre-Clinical Assessment

The safety of dietary supplements is generally evaluated using both *in vitro* and *in vivo* studies. One particular *in vitro* study compares the chemical structure of the supplement to that of a compound known to be toxic. If the supplement’s structure is similar to that of a known toxin, testing of the compound ceases. If, however, the compound does not appear to be toxic, it can then be evaluated using the Ames test [62]. This test uses a defective strain of Salmonella typhimurium that proliferates if it is exposed to a toxin. This is not a perfect test because not all toxins increase the proliferation of Salmonella typhimurium, and it does not predict how the compound will affect eukaryotic cells. Furthermore, the Ames test does not provide any information on whether the compounds own metabolites are toxic.

*In vivo* studies provide information regarding the safety of the compound in the supplement but also the safety of the compound’s metabolites. They also provide pharmacokinetic and pharmacodynamic information regarding the supplement. Most companies perform studies using more than one animal species. Animal studies include, but are not limited to: a 90-day feeding study during which the animal receives very high doses of the supplement; a two-generation feeding study which determines if the supplement is likely to have any adverse effect on the next generation of the animal ingesting the product; and long-term studies that last more than two years.

## 8. Utility and Limitations of Clinical Trials

Herbal supplement manufacturers may decide to conduct human-based clinical trials, but are not legally obligated to do so, unlike pharmaceutical drug manufacturers who must legally complete such trials. Clinical trials are used to determine the pharmacokinetics, pharmacodynamics, and safety of the drug. They can also identify specific subpopulations that may be adversely affected by a drug.

In the case of prescription products, the results of clinical trials performed by sponsors play a central role in characterizing the efficacy and safety of drugs and biological agents and are a backbone in the FDA’s consideration of their approval for marketing in the US. With the lack of validated pre-clinical models or markers to reliably identify all agents that have a potential to cause DILI in humans, clinical trials have also proven to be an invaluable tool for the identification and characterization of hepatotoxic risk of new agents during their development, both by the pharmaceutical industry and by FDA regulators [63,64,65]. As described above, with just a few exceptions, in which herbal products have been approved for the treatment of specific disease indications by FDA, this avenue for hepatotoxic risk assessment of new dietary supplements by regulatory scientists prior to their marketing is typically not available.

The systematic and periodic biochemical and clinical monitoring of all study subjects and comprehensive assessment of each case of liver injury that occurs in trials offers an opportunity to identify critical profiles of hepatotoxic risk (often referred to as “signals”) that are characteristic of both the specific study agent as well as individuals who are susceptible to injury in the study population [65]. As fully described in a 2009 FDA Guidance for Industry (entitled: “Drug-induced liver injury: premarketing clinical evaluation”) [66], the assessment of these signals within the study populations is a particularly important approach to predict risk for clinically serious DILI in a large post-marketing population that will later be exposed to the same agent. In clinical trials, there are two types of liver signals of especial importance. First are cases of serious liver injury marked by hepatocellular necrosis and/or apoptosis leading to a reduction in liver function. Hy’s law cases are in this category [67,68]. They are defined as cases with new onset acute rises in serum alanine aminotransferase (ALT) (and usually aspartate aminotransferase (AST)) values together with new elevations of serum bilirubin levels (in some instances, international normalized ratio (INR) levels also are raised) that are causally related to the study agent; this requires that all plausible alternative etiologies of acute liver injury have been systematically ruled out, including acute viral hepatitis, possible injury from concomitant drugs, heart failure, acute hypotension, choledocholithiasis, *etc*., and additionally, if supplements are implicated, possible exposure to contaminants and adulterants. The presence of one or more Hy’s law cases indicates that there is a strong likelihood that idiosyncratic serious liver injury cases will occur with the same frequency in a similar large post-market exposure population. A percentage of cases of serious DILI (10%–50%) may progress to acute liver failure, while some might advance to cirrhosis and/or chronic liver dysfunction.

A second scenario is an increase during randomized clinical trials of ALT levels but not of serum bilirubin values showing an imbalance between those treated with the study agent and those who had received the comparator or placebo. This imbalance is consistent with possible emerging DILI, but alone, without cases of more serious injury in the study population, does not provide a quantitative prediction (or exclusion) of risk in a large post-market treatment population for serious liver injury; mild injuries caused by some agents will almost always be self-limited and not accelerate to more serious damage. This is due to universal cytoprotective and adaptive mechanisms that operate in the liver.

As useful as clinical trials are to identify hepatotoxic drugs, they are limited in a number of ways. Serious liver injury marked as Hy’s law may occur with an incidence of only 1/1000 or even less frequently among study subjects treated with some idiosyncratic hepatotoxins. To exclude a risk for hepatotoxicity in 1/10,000 users of the agent that results in serious liver injury, taking into account the statistical rule of three (95% confidence interval), requires that approximately 3300 study subjects must be exposed to the agent without this event occurring [69]. Thus, the ability to predict rare idiosyncratic hepatotoxic events from clinical trials is limited by the powering of the study. Also, if the risk of hepatotoxicity increases only after a minimal exposure period, or the threshold of cumulative dosing has been exceeded, the use of short term treatment protocols may not uncover liver injuries that are only associated with chronic use of the study agent. Because of these limitations in short-term clinical trials, the detection and assessment of the hepatotoxic risk from therapeutic and supplement products also depend on post-market case reports and other surveillance and epidemiological methods.

Although not subject to FDA’s investigational new drug regulations, there are a rising number of clinical studies of herbal products and dietary supplements that have been registered with the web-based NIH registry of clinical trials (clintrials.gov) [3,70]. Analyses of data from trials of some herbals suspected from post-market reports of causing hepatotoxicity have been performed to determine if the trial results are consistent with the reported cases. For example, a meta-analysis of black cohosh trials did not confirm concerns about hepatotoxicity generated from a number of published post-market case reports of toxicity associated with this botanical agent [71]. In the future, with ingredient and contaminant fingerprint identifiers archived in the record, analysis of such trials for hepatotoxicity signals will gain greater traction in the academic health and nutrition communities.

## 9. Characterization of HDS Product Chemical Content

From about the mid-1990’s, a number of academic experts have engaged in studies to elucidate the efficacy and safety of herbal supplements, demonstrating the importance of using different HPLC-based systems to authenticate and standardize agents before their testing in pre-clinical and clinical studies [72]. Supplement fingerprinting using advanced chromatographic and spectroscopic techniques would support their integrity and enhance the comparability and analysis of results across different studies. Recently, DNA fingerprinting of herbal ingredients has also been applied as a useful academic tool to authenticate plant species that are present in study supplements [28]. In alignment with the scientific merits of chemical or DNA fingerprinting, the NCCIH has established a policy that investigators who perform a government-funded clinical study of a botanical supplement should accurately identify the taxonomic nomenclature of the source plant(s), provide data on its chemically fingerprinted profile using the aforementioned techniques, together with certificates of analysis from suppliers, batch-to-batch reproducibility, solvents used for extraction, contaminants and product stability [73]. In the private sector, there are a number of organizations that perform analyses of production and content of dietary supplements. For example, the US Pharmacopeia has established a Dietary Supplement Verification Program to which manufacturers can submit products for production analysis, testing and identification of contents [74].

A diverse set of chromatographic and spectral tools, as well as DNA fingerprinting methods have been used for the authentication of herbal products and identification of botanical species [28,75]. Metabolite profiling of serum has also been used to document ingestion of specific phytochemicals. Often, more than one chromatographic system must be utilized to comprehensively separate and analyze all the phytochemicals that are present in a complex herbal mixture. Methods used for this purpose include HPLC and UHPLC, GC and TLC [76]. Spectral methods used for botanical analysis include near infrared and UV spectroscopy and NMR [77]. Over time, with the development of databases that comprehensively archive fingerprints of each botanical species, reliable protocols for accurate identification of botanical components in complex herbal supplements are expected to gain traction for use by manufacturers, as well as regulatory and public health scientists.

## 10. *In Silico* Modeling of Drugs and Other Agents that Cause Idiosyncratic Hepatotoxicity

There has been a growing interest among academic experts, drug developers and regulatory scientists to develop algorithms that would reliably model and predict the risk for hepatotoxicity associated with specific drugs and biological agents. Such models integrate information about the chemical structure/function characteristics, pharmacological actions, pharmacokinetics, metabolism and clearance of the treatment agent. They incorporate assumptions about the physiological, biochemical, cytoprotective and regenerative responses of liver cells to predict threshold conditions that will precipitate clinically serious hepatotoxicity as well as the incidence and outcomes of these injuries [78]. Environmental, genetic and other factors that increase “outlier” susceptibility to idiosyncratic DILI can also be simulated as modules inserted within a larger model to predict effects of inter-individual variation on population-based risk. In building these models, iterative refinements and improvements should be made by “best-fit” adjustments with empirically derived clinical and animal or cellular datasets.

Although a number of DILI *in silico* modeling projects show promise, they currently are early in their development as research tools only [78]. To date, assumptions made in these models hinge primarily on pre-clinical and clinical data collected with a few extensively studied hepatotoxins, especially acetaminophen over-dose. Dosage effects and individual susceptibility conditions that instigate a critical loss of hepatocellular mass and serious hepatotoxicity with acetaminophen exposure are known to be distinct in many respects from those linked to many other hepatotoxins that cause idiosyncratic injuries. One of the challenges for further improvements in the developing DILI models is a need to incorporate multiple modules that account for each of a wide variety of different mechanisms that underlie hepatotoxicity. Such models have yet to be developed sufficiently and widely applied as tools for the prediction of idiosyncratic botanical-induced hepatotoxicity.

## 11. Post-marketing Assessment of Herbal Hepatotoxicity

### Surveillance Databases and Tools

A number of databases exist allowing identification of adverse events attributable to dietary supplements, including herbal products. These include two FDA spontaneous report databases, one housed in FDA’s Center for Food Safety and Applied Nutrition (CFSAN) (the CFSAN Adverse Event Reporting System (CAERS)) and the other in FDA’s Center of Drug Evaluation and Research (the FDA Adverse Event Reporting System (FAERS)); the National Electronic Injury Surveillance System (NEISS); and the American Association of Poison Control Center.

CAERS is the database that accepts both mandatory and voluntary reporting of dietary supplement adverse events. Mandatory reporting is required of supplement manufacturers who must submit a MedWatch form within 15 business days of a serious adverse event notification. The MedWatch report must contain information about the reporter, the injured party, the product, the adverse event, and the manufacturer of the supplement. Voluntary reports usually come from consumers or health care providers and may consist of a serious or non-serious adverse event. A serious adverse event is defined as a “death, a life-threatening experience, in-patient hospitalization, a persistent or significant disability, congenital anomaly, or requires, based on reasonable medical judgment, a medical or surgical intervention to prevent an adverse outcome” [79]. FAERS receives post-marketing adverse event and medication error reports associated with drugs and therapeutic biologic products [80]. Dietary supplement-associated adverse event reports that involve drug adulterants or concomitant therapeutic products are entered into FAERS.

An FDA clinical scientist reviews every serious dietary supplement adverse event and can decide to investigate the event in more detail by requesting follow-up information from the submitter. The review approach may simply be to focus on a single serious adverse event associated with a dietary supplement and then to search the medical literature to determine if the supplement had been implicated in the past in causing liver injury. Alternatively, it might utilize a data analysis program, which reviews the data and informs the reviewer of any unusual patterns regarding the safety of a product. The FDA clinical scientist, in conjunction with statistician colleagues, will then determine whether the data analysis program has identified a pattern of adverse events associated with a product or whether it has detected a false signal.

The value of CAERS, if properly populated, is that it provides data needed for determining whether the adverse event was likely associated with the ingestion of the product. Also, since it includes the reporter’s contact information, the reporter can be approached to obtain additional information as necessary. Unfortunately, adverse event reports are quite often incomplete and are not always submitted as required.

The National Electronic Injury Surveillance System (NEISS) supports the Consumer Products Safety Commission (CPSC) and “is a national probability sample of hospitals in the U.S. and its territories. Patient information is collected from each NEISS hospital for every emergency visit involving an injury associated with consumer products”. The information that can be obtained from NEISS includes: demographic characteristics of the population injured, the product associated with the adverse event, and treatment that the consumer received in the emergency department. The CPSC can use this information to take regulatory actions, as needed, with reference to products it regulates or it can forward the information to another regulatory agency such as the FDA. A shortcoming of this database is that follow-up information is usually not obtained.

The third database is the National Poison Data System, maintained by the American Association of Poison Control Centers. Healthcare providers and consumers can contact a Poison Control Center (PCC) and receive guidance that will help them manage the consumer’s condition. In 2013, PCCs received over 3.1 million calls. Data captured by this system are: the consumers’ demographic characteristics and whether the consumer was treated at home, by a healthcare provider, or in an emergency room. The PCC is an effective system since it is utilized by large numbers of consumers. As with CPSC above, the shortcoming of the system is that it collects very little follow-up data about the affected consumer.

One hurdle facing pharmacovigilance programs is the difficulty in merging all the accumulated data. Integrating CAERS, NEISS and PCC data into one database would be a step in that direction. Another would be the ability to map each individual system to align with the corresponding fields of the other databases.

## 12. Challenges in Assessing Suspected Herbal Hepatotoxicity

Finding a link between exposure to a herbal supplement and hepatotoxicity depends on a comprehensive assessment of both the clinical and diagnostic features of post-marketing cases of liver injury of concern as well as a full accounting of the suspect product and its hepatotoxic profile [81]. In order to establish a causal association with a supplement ingredient, chemical, adulterant or other contaminant, a series of issues must be considered. First, bona-fide cases of liver injury causally tied to herbal supplement exposure may go unrecognized or may be inaccurately or incompletely reported to the CFSAN’s CAERS. Highly informative reporting of these events requires that information be provided regarding the clinical and biochemical nature of the liver injury event and a full accounting of data obtained to systematically rule out all plausible causes of the liver injury other than the supplement exposure [81]. Clinicians need to be aware that DILI caused by different hepatotoxic agents may have distinct pathological and clinical profiles. Patterns of liver injury, when acute, may be predominately hepatocellular or cholestatic; when chronic, they may result in cirrhosis or veno-occlusive disease [68]; Second, botanical commercial products are often comprised of concentrated and complex chemical extracts that are derived from many kinds of plants. Because they come from plant parts that are selected and prepared from different cultivations and batches of manufacture, it is inevitable that there would be some variability in product phytochemical content of seemingly similar or identical herbal components and protocols of manufacture; Third, there is no comprehensive archive of product-specific chemical fingerprints that is accessible to regulatory scientists for routine use as a basis of comparison when analyzing the fingerprint of a product unit directly linked to a post-marketing case of liver injury. Absence of such standards would generally preclude the rapid identification of candidate culprit hepatotoxin “peaks” from complex chromatographic, spectral or other signatures of herbal formulations. Contaminants, adulterants or levels of molecular oxidation may alter the quality of herbal products or other chemical modifications not accounted for in many molecular fingerprinting methods; Fourth, dietary supplement manufacturers have been inconsistent in submitting NDIs prior to the marketing of new products in the US. This inconsistency is borne out anecdotally by recent product withdrawals due to lack of FDA notification surrounding ingredients that should have been considered NDIs (see Section 14). Of equal concern, between 1995 (when NDIs first were required) and 9 July 2015, the FDA received only 725 notifications, a small fraction of the more than 50,000 dietary supplement products marketed in the US in that same period [82]. This suggests that chemical agents and other new ingredients that have been added to dietary supplements only in recent years may often go unreported as NDIs.

Despite these concerns, as described below, there has been remarkable progress in the development of advanced analytic tools to quantitatively characterize the chemical and botanical composition of dietary supplements, as well as any contaminants and adulterants that may be present. These have a growing role in the identification of misbranded products, to enable evaluation of products associated with adverse events, such as suspected hepatototoxicants, and/or facilitate scientifically sound corrective regulatory or public health interventions. For example, on 24 February 2015, the Attorney General of New York State ordered four major retailers of healthcare products and pharmaceuticals to remove certain store-brand herbal products from their shelves [83]. He also demanded that their manufacturers provide information on the processes of production and the ingredients contained in each of their products sold in the state. This action was taken because of results of DNA testing performed on behalf of state authorities that showed poor correspondence between the listed botanical ingredients on the product labels and the measured contents in the purchased products. The value of advanced analytic tools to identify the misbranding of dietary supplements extends beyond the authentication of botanical ingredients [28]. Scientific methods to screen for unlawfully added adulterants including prescription drugs and controlled substances, some of which have been linked to DILI, have proven to be very important tools for regulatory scientists. In multiple instances, pharmacologically active adulterants have been identified in dietary supplement products marketed in the US to improve muscle strength, induce weight loss, enhance sexual performance, or boost energy. Examples of drugs that have been identified through such screening are described in detail below.

## 13. Causality Assessment

There have been numerous reports over the years of herbals implicated in the occurrence of hepatotoxicity. Initially, these had involved the individual traditional herbals, but increasingly, commercially produced multi-ingredient products are being implicated, and now represent the bulk of reports of herbal hepatotoxicity [1,2,3,84,85,86]. Of note is that some traditional herbals not known in the past to have caused liver injury have more recently been implicated as a cause for DILI, believed to be a result of newer methods used to extract the active ingredient; an example, as has already been noted, is that Kava–Kava appears less hepatotoxic when subjected to aqueous extraction than when extracted by organic solvent fractionation [56].

The true frequency of hepatotoxicity from herbals is unknown. Not surprisingly, relative to all identified cases of DILI, the proportion attributed to botanicals is extremely high in Asian countries, 73% in Singapore [87] and 71% in Korea [88], but is far lower in Western countries. In three large surveillance databases (US, Spain, Iceland), hepatotoxicity attributed to herbals accounted for from 2% to 20% of all identified cases of DILI, including both pharmaceutical drugs and herbals [7,89,90,91]. Data in the US study suggested that the proportion of herbal-related DILI cases appeared to be increasing relative to all identified cases of DILI [7].

In conjunction with the hepatotoxic profile of the specific drug, biological agent or herbal in question, dosing effects or individual patient susceptibility are major determinants in predicting the risk for the development of DILI. Drugs that cause hepatotoxicity typically fit on a spectrum between those that are “direct” hepatotoxins and those in which the toxicity is “indirect”. A predictable dose or exposure threshold marks direct hepatotoxins, when risk for liver injury rises quickly for most exposed individuals. Examples of herbals whose extracts are directly toxic when ingested at high exposure levels include Symphytum officinale (Comfrey), Crotalaria, Heliotropium and Senecio [84]. These plant species contain a number of different pyrrolizidine alkaloids which, when ingested in high amounts, cause severe toxicity through a mechanism of hepatocellular biotransformation into genotoxic pyrrole derivatives. The most common form of liver injury caused by these products is the sinusoidal obstruction syndrome that is marked by non-thrombotic obliteration of the hepatic sinusoids and terminal centrilobular hepatic veins (see Section 13). Indirect hepatotoxins cause significant liver damage in only a fraction of those who are exposed to the agent because of the impact of susceptibility factors. It is important to note that even the threshold for “idiosyncratic” toxicity in these individuals can be influenced by exposure to other drugs and modified by a number of environmental and genetic variables. Although HLA allelic susceptibility marker associations with hepatotoxicity linked to some drugs point to adaptive immunity as having a central role in idiosyncratic DILI, other mechanisms including the formation of excessive toxic drug metabolites, hypersensitivity and mitochondrial damage also have been linked to certain hepatotoxic drugs [92]. With different drugs connected to a number of distinct pathological mechanisms that are the root cause of the injury, predicting risk for DILI in supplement users is an especially difficult exercise for regulatory scientists.

When regulatory scientists assess cases of possible idiosyncratic supplement-associated liver injury that occur in a post-market setting, there often are many plausible explanations that must be considered regarding identification of the real event and pinning down the true toxic chemical moieties or components in the formulation. A sizable number of different herbal species have been associated with reported cases of hepatotoxicity that in some instances are connected to known direct or indirect biological mechanisms of liver injury. Misidentification of the substance “in the mix” that is responsible for the liver injury can lead to incorrect conclusions about the nature of the event and misconceived regulatory actions intended to prevent further cases of liver toxicity. For example, if the culprit responsible for liver damage is an adulterant or toxic contaminant (e.g., aflatoxin, heavy metals, new ingredients that are hepatotoxic) that in some instances may be present in some but not all batches of the product, there may be a public health hazard which would require quick regulatory action (exemplified in the case of OxyELITE Pro (see Section 14). On the other hand, if liver toxicity stems from a rare idiosyncratic immune-mediated reaction to a labeled herbal ingredient with dosing that is generally considered safe, further evaluation of usage patterns and adverse events in the population might be justified. There is no obvious reason to think that many of the drug-associated mechanisms that underlie serious idiosyncratic DILI would not apply to phytochemicals in dietary supplements. However, other types and causes of liver injury that are linkable to dietary supplements in particular must be accurately excluded. Unfortunately, identifying the root cause of hepatotoxicity and pinpointing the actual moiety that is responsible in complex supplements whose ingredients are not initially authenticated remains an enormous challenge. This challenge is compounded by the usual absence of clinical safety data reviewed in a systematic fashion by regulatory scientists prior to the marketing of most new supplement products. Furthermore, the hepatic effects of excessive or long-term continuous dosing in a large exposure population are often uncharted when the marketing of a new product is initiated.

As described above, the actual contents of herbal products, especially the multi-constituent commercial products, are complex and can vary considerably in terms either of their concentration or their actual contents. In some instances unlabelled pharmaceutical products may be added (adulteration), and there may have been exposure to chemical or biological contaminants. Thus the dilemma in diagnosing herbal-related DILI is not so much its attribution to an administered herbal, but rather the identification of the responsible “hepatotoxic” constituent, whether the herbal itself or a contaminant/adulterant. Accordingly, identifying the responsible agent requires analytic measures, a difficult task and not yet standardized.

### 13.1. Liver Toxins

Liver injury, whether from pharmaceutical drugs or herbal products, occurs either as an idiosyncratic reaction or as direct toxicity. With regard to botanicals, direct hepatotoxicity may be the result of the toxic element in the herbal itself, or may result from contamination of the plant in the process of harvesting. A prime example of the former occurrence is the presence of pyrrolizidine alkaloids in a number of botanicals including monocrotaline, crotolaria, heliotropium and Simphytum officinale (Comfrey) [84]. With the exception of Comfrey that has been used for medicinal purposes, toxicity from the other products comes mainly from their contamination of crops and foodstuffs [93,94,95,96,97]. The injury—sinusoidal obstruction syndrome—is clearly a dose-related phenomenon [98]. Other examples of herbs that can cause direct liver injury are green tea used in high doses, regarded to be a result of the presence of epigallocatechin gallate (EGCG) and epicatechin gallate [99,100,101,102]; Germander due to the presence of dipteroids [103]; Chaparral, attributed to nordihydroguaiaretic acid [104,105]; Atractylis gummifera [106]; and Callilepsis laureola [107].

Contamination of botanicals, unlike adulteration, is generally an unintentional event that results from growing, spraying and harvesting of the parent herbal plant. Numerous reports, often involving products sold over the internet, many from China or India, list contamination with pesticides [12,13,17,108,109].

Perhaps the best known and best described of the natural toxins are the aflatoxins (Aspergillus flavus, Aspergillus parasiticus) [110]. Aflatoxins can contaminate maize, peanuts, rice and other crops, causing an acute toxic hepatitis associated with a high mortality [111,112]. The aflatoxins are also carcinogenic, well known to cause hepatocellular carcinoma, but also carcinoma of the kidneys, large bowel, and gallbladder [113,114,115,116,117,118].

### 13.2. Drug Adulterants with Known or Possible Hepatotoxic Profiles

An important category of liver injury caused by exposure to both herbal and non-herbal preparations is hepatotoxicity caused by an adulterant(s) [72,119,120,121,122,123,124,125,126,127,128,129,130,131,132,133,134]. Adulterants that should not be present in dietary supplements may be introduced intentionally or unintentionally into the supply chain at any step between the planting phase and production and packaging phase of the marketed formulation. It is noteworthy that occasional unintentional cross-contamination with drugs or pharmacologically active agents related to poor manufacturing practices has occurred [119]. However, the vast majority of adulteration cases in this category are due to intentional manipulation. When adulteration with unlabeled prescription or over-the-counter drugs or controlled substances is intentional, it is typically driven by a desire to increase or alter the claimed effect of the marketed product to gain a commercial advantage. Various methods can be employed by regulatory scientists to screen for adulterants. Depending on the drug substances identified in these screens of supplements, the techniques that have been used include Liquid Chromatography-MS, IR Spectrometry, Gas-Chromatography-MS and Ion Mobility Spectrometry (IMS) [72,122,123,124,125,126,127,129,130,131,135].

In weight loss supplements, drug adulterants that have been identified include sibutramine, fenflurane and phenolphthalein [126,127]. All of these drugs have been removed from the US market due to safety concerns. Other adulterants that have been identified in the weight-loss products include diethylpropione, 1,3-dimethylamine (DMAA), fenproporex, furosemide, rimonabant, and cetilstat [119]. In the case of sexual performance enhancing supplements, high rates of product adulteration with phosphodiesterase-5 inhibitors, including sildenafil, tadalfil and vardenafil have been observed [119,120,121,122].

Not surprisingly, muscle-building supplements have often been found to be adulterated with anabolic steroids or aromatase inhibitors, both classified as prescription drugs. Anabolic steroids are generally classified based on their biological effects that are mediated through the binding and activation of the androgen receptor. The prototype androgen is testosterone, which can be detected easily by immunoassays or spectrophotometric analysis. In contrast, “designer steroids” discussed further below, are *de novo* synthesized androgenic compounds that have structural similarities to testosterone but can be more difficult to detect or analyze by standard chromatographic or spectroscopic techniques. With the exception of dehydroepiandrosterone (DHEA), most anabolic steroids are designated as Schedule-3 controlled substances. Hepatotoxicity continues to be reported as a consequence of consumer usage of supplements that are illegally spiked with anabolic steroids.

The Division of Pharmaceutical Analysis (DPA) in CDER has developed analytic and quantitative protocols to screen samples of marketed products for their quality or for the presence of drug adulterants [136]. The division’s laboratory expertly utilizes methods including GC-MS, Accurate LC-MS, HPLC with diode array detection, and UV and NMR Spectroscopy to screen for many different adulterant drugs and other hidden substances in suspected products under investigation, including dietary supplements. The laboratory has developed protocols to fractionate new potentially anabolic steroids which may have hepatotoxic potential from complex mixtures using GC or LC MS techniques and characterize them chemically using spectroscopy.

## 14. FDA Regulatory Actions for Hepatotoxic Supplements: Anecdotal Examples and Experience

The FDA has taken regulatory actions over the course of some years regarding several herbal products. Examples are supplements marketed as weight loss products or for use by athletes and body builders for muscle enhancement.

### 14.1. Lipokinetix (Usnic Acid)

Because the FDA had received several adverse event reports of acute hepatitis and/or liver failure after consumers had used Lipokinetix [137], the FDA, in November 2001, issued a “Dear Health Care Provider” letter regarding its safety [138]. What was unique about Lipokinetix was that it induced liver injury in individuals between 20 and 32 years of age who had no history of liver injury. Lipokinetix was promoted for weight loss by mimicking exercise and supporting an increased metabolic rate. To cause this effect, the product contained norephedrine, caffeine, yohimbine, diodothyronine, and sodium usniate. Usnic acid was believed to be the ingredient that caused liver injury [139,140].

Usnic acid is a compound found in the usnea species of lichens [141]. It has been used in perfumery, creams, toothpaste, mouthwash, deodorants and screens. *In vitro* studies have demonstrated antiviral, antiproliferative, and anti-inflammatory activities. Usnic acid has also been studied in clinical trials to treat genital human papilloma virus using an intravaginal suppository. Sixty-five cases of Tinea pedis improved after the patients were treated with topical usnic acid.

Usnic acid's most common side effects are local irritation and dermatitis, particularly when applied to the skin. An increase in oxygen consumption and hyperventilation has been observed in anaesthetized cats that received a dose of 10 mg/kg. A study in mice demonstrated that usnic acid interferes with liver mitochondrial function [142]. No clinical trials have been done to determine toxicity in human subjects. However, based on the findings of the aforementioned animal studies and the fact that Lipokinetix caused liver injury in consumers who used it, it is reasonable to conclude that usnic acid was the cause of liver injury. After FDA posted its “Dear Healthcare Provider” letter, Lipokinetix was removed from the market.

### 14.2. OxyELITE Pro

In 2012, the manufacturer of OxyELITE Pro was informed by the FDA that its formulations that contained DMAA, an ingredient linked to cardiovascular abnormalities such as tachycardia and hypertension [143], had to be removed from the market or reformulated without it. Accordingly, the company removed the products that had included DMAA. Later, two reports, one from Hawaii and the other involving active duty service members, described cases of acute hepatocellular injury from presumably DMAA-free OxyELITE Pro; some had required liver transplantation [144,145]. On 19 November 2013, the FDA informed the public that the new OxyELITE Pro formulation had been associated with liver adverse events [146]. Seventeen patients had used OxyELITE Pro alone at the time of the adverse event. One consumer had required a liver transplant, and several others were awaiting liver transplantation at the time of the reports. Because of these findings, there was heightened concern by public health authorities from the CDC and FDA regarding the likelihood of a causal or contributory link between the ingestion of the formulation of OxyELITE Pro containing aegeline and some cases of liver injury [147,148,149,150]. It must be noted, however, that Teschke and colleagues, in reviewing the case material that they were able to obtain from the Hawaiian cases, strongly questioned the adequacy of interpretation of the liver injury cases and the validity of establishing any causal association with OxyELITE Pro [151,152].

Since the FDA received an increasing number of MedWatch reports in 2013 associating OxyELITE Pro with liver injury, submitted from different US sites both on the US mainland as well as from Hawaii, a decision was made to obtain and chemically analyze samples of the product. The FDA’s Forensic Chemistry Center analyzed 18 samples of OxyELITE Pro; thirteen were obtained from patients who experienced liver injury and the remaining samples were obtained from retail shelves. The results of the analysis demonstrated that many of the tested products represented a new formulation, containing the combination of aegeline, higenamine, caffeine, and yohimbine [147].

After obtaining the results of the chemical analysis, the FDA sent a letter to USPLabs informing them that one of the ingredients of concern in the reformulated OxyELITE Pro was aegeline. Aegeline had never been present in a US-marketed dietary supplement prior to 1994, and USPLabs had not filed an NDI notification informing FDA that it was safe. Since its presence was apparently associated with numerous liver and other serious adverse events, the supplement was classified as a risk to public health and was therefore removed from the market. If USPLabs had not instituted a voluntary recall of OxyELITE Pro from the market, the FDA could have used its authority granted under the Food Safety Modernization Act to mandate that USPLabs stop manufacturing and distributing OxyELITE Pro, and that it must also inform other parties that they could not distribute OxyELITE Pro [147].

Most recently, a new product termed OxyELITE Pro Super Thermogenic, has been found to contain fluoxetine (Prozac), an antidepressant that is associated with potentially serious side effects. Once again, the FDA issued a public advisory informing consumers not to purchase this product [153].

### 14.3. Hydroxycut

Hydroxycut was introduced to the market in 2002 (Iovate Health Sciences Research, Oakville, ON, Canada) as a weight loss supplement. Billed as a “fat burner”, it was advertised over the Internet and sold in retail chains stores. Shortly thereafter, the first report appeared of two cases of acute hepatocellular injury, associated with deep jaundice; both patients recovered [139]. The original formulation contained ma huang (ephedra), a substance that was banned by the FDA in 2004 because of its association with cardiovascular, neuro-psychiatric, and gastrointestinal side effects [154]. Accordingly, the manufacturer removed ephedra from the formulation, but cases of DILI continued to occur. Thus far, almost 20 cases of liver injury from this product have been reported, among whom a number have had to undergo liver transplantation [155,156,157,158,159,160,161,162]. Most cases have presented as hepatocellular liver injury although a few developed cholestatic injury [156,160]. The constituents of Hydroxycut include the following: calcium, chromium, potassium, hydroxagen plus, *Garcinia cambogia extract*, *Gymnena sylvestre extract*, soy phospholipids, *Rhodiola rosea extract*, *Withania somnifera extract root*, hydroxy tea, Green tea extract (*Camellia sinensis*), White tea extract, Oolong tea extract, and Caffeine anhydrous. Which of these components is the specific cause for the liver injury is uncertain, but suspicion has fallen on *Camellia sinensis*, that has been implicated in the past in causing liver injury [101,102,163]. In view of the numerous reports of Hydroxycut DILI, the FDA warned the public of the severe risk of liver injury attributable to the herbal product, and the manufacturer withdrew it from use [164]. However, Hydroxycut returned to the market with a different formulation, entitled Hydroxycut, SX-7 Clean Sensory, despite which a new case of liver injury has been reported [165].

### 14.4. Designer Steroids

Anabolic-androgenic steroids (AAS), developed in the 1930s, are used to stimulate muscle growth and therefore have long been used by athletes to improve fitness and exercise performance. However, because of a number of clearly identified adverse health effects from these products, the US Government elected to place them under the Controlled Substance Act, the Anabolic Steroids Control Act of 1990, listing a number of AAS products made illegal by the Act [166]. Public concern later of the safety of prohormone precursors of testosterone prompted their classification also as class III substances through enactment of the Anabolic Steroid Control Act of 2004, thus essentially banning the use of these products in the US [167]. In order to circumvent controlled substance laws, new forms of anabolic steroids began to be developed, synthesized and modified from a parent steroid, thus referred to as “designer steroids” [168,169,170,171]. These synthetic anabolic steroids then were added to and sold as dietary supplements, but their presence was not shown on the label. Directed to athletes for improving sport performances, they are marketed as alternatives to anabolic steroids for increasing muscle mass and strength. In 2009, the FDA issued a public health advisory warning consumers that some products marketed for bodybuilding and claiming to contain steroids or steroid-like substances are illegal and potentially dangerous [172]. A warning letter, sent to American Cellular Laboratories, Inc. listed a number of products of concern, including “TREN-Xtreme”, “MASS Xtreme”, “ESTRO Xtreme”, “AH-89-Extreme”, “HMG Xtreme”, “MMA-3 Xtreme”, “VNS-9 Xtreme” and “TT-40-Xtreme” [173]. More recently Congress enacted the Designer Anabolic Steroid Control Act of 2014, expanding the list of anabolic steroids to be regulated by the Drug Enforcement Administration (DEA) that included about two-dozen new substances [174].

Anabolic steroids are well known to cause various forms of liver disease [175] including intrahepatic cholestasis [176,177,178], hepatocellular carcinoma [179,180,181], adenoma [181,182], and peliosis hepatis [183,184]. Despite the fact that anabolic/androgenic steroids are classified as class III controlled substances, they continue to be available as designer steroids, often through the internet, and continue to be associated with the various forms of liver disease. This includes cholestatic liver injury [185,186,187,188,189,190,191,192], hepatocellular carcinoma [193], adenoma [194,195,196], and peliosis hepatis [197]. In the multi-center NIH-sponsored US prospective DILIN study, approximately 5% of patients who were referred to the network with DILI developed liver injury in association with the use of bodybuilding supplements [7]. In a collaborative study in the United Kingdom (UK) led by investigators in the King College Drug Control Center, body-building products that were purchased in two fitness equipment shops were analyzed with gas and high pressure liquid chromatography, NMR and X-ray crystallography enabling the accurate identification of anabolic steroid compounds [198]. Strikingly, 23/24 of the products that were tested contained steroids; 16 of these were different from those displayed on the packaging. Overall, thirteen different steroids were identified, including 12 that are controlled substances in the UK. As designer steroids, many of these were not previously known to be commercialized. Recently, there has been a report of the development of toxicant-associated fatty liver disease (TAFLD) in male bodybuilders using anabolic-androgenic steroids, a condition previously attributed to industrial toxins, and equivalent to the metabolic non-alcoholic fatty liver disease (NAFLD) [199].

## 15. Global Regulation of Herbals and Dietary Supplements

While the use of traditional medicine has existed for millennia among developing countries, its use in more developed countries has been more recent, but is growing exponentially. Currently, the market for herbals and dietary supplements in the US is estimated to have a value of $62 billion, and the World Health Organization (WHO) projects that this will increase to $5 trillion by 2050 [200]. Despite the worldwide expansion of the use of botanical products, the WHO found that among the 191 listed member countries, only 25 had a national policy regarding herbals and only 64 regulated them [200]. Accordingly, in 1998, the WHO developed and published technical guides and regulatory policies on botanicals as an aid for the various countries [201]. Since then, there has been a general increase in the attention paid to the issue, although the approaches instituted have varied among the countries. A major discrepancy relates to definitions of what constitutes a foodstuff, a supplement or a medicine since in most instances, these are regulated differently [201]. In 2005, the WHO published the results of a worldwide survey of the approaches taken by member states for the regulation of herbal medicines [22,202].

## 16. Enhancing Research in the Evaluation and Management of Herbal Hepatotoxicity: Future Directions

As has already been noted, identifying the specific moiety in an herbal product implicated in causing liver injury has represented a major hurdle. Not only is there the possibility that an untainted single botanical may vary from lot to lot depending upon the growing conditions at the time of harvesting so that its constituents may differ in quality and quantity, but there is always the concern that there may be accidental contamination or adulteration. This poses the problem of establishing whether the liver injury was a result of direct or idiosyncratic toxicity and what the precise constituent was that led to the liver injury. Now that there is clear evidence of expanding use of herbals and dietary supplements, and with the growing realization that some of these products have been responsible for causing DILI, increasing numbers of scientists and investigators are turning their attention to new approaches to improve the management of these risks. First, expanding the use of precision chromatographic, spectroscopic and other analytic methods for the comprehensive chemical characterization of products suspected of causing hepatotoxicity would be an important step to improve risk assessment [203,204]. Undoubtedly the most effective means of conducting such investigations would be to have ready access to the actual product(s) implicated in causing liver injury. This approach is presently ongoing in the US DILIN study [8]. Second, the development of publically accessible comprehensive databases containing the signatures of non-hepatotoxic herbal products using these techniques would be an important set of resources for scientists and investigators. They would serve as a frame of reference in analyzing the chemical profiles of products linked to hepatotoxicity. As discussed above, a Department of Agriculture sponsored initiative to pilot the feasibility of cataloging one category of herbals in such databases is a promising step in this direction.

Third, the future of post-marketing adverse event analysis lies in the ability to process large amounts of global data quickly. Unfortunately many centers that collect adverse event data are not digitally interconnected and therefore cannot promptly share their data. Other obstacles that must be overcome are that many of the adverse events monitoring systems obtain little information regarding adverse events, and many individuals who experience an adverse event(s) have more than one medical condition and/or are taking more than one drug.

To overcome these limitations adverse event data collecting centers must agree to share their data and make them broadly accessible to health care providers, public health scientists and researchers. Although a great number of reports will not be able to link an adverse event to a product, modernized adverse event database analysis programs could provide these investigators with a set of search and filtering tools to facilitate the effective and timely triage of liver injury cases of interest.
